# Long term culture of MDCK strains alters chromosome content

**DOI:** 10.1186/1756-0500-6-162

**Published:** 2013-04-24

**Authors:** Doris Cassio

**Affiliations:** 1 Inserm UMR-S757, Orsay, 91405, France; 2 Université Paris-Sud, Orsay, 91405, France

**Keywords:** Madin-Darby Canine Kidney (MDCK) cells, MDCK I, MDCK II, Metaphase, Chromosome content, Cell line drift

## Abstract

**Background:**

The Madin-Darby Canine Kidney (MDCK) cell line and its different strains are widely used as models for studying epithelial simple polarity. Recently Dukes et al. [BMC Cell Biology 12:43, 2011] provided a useful guide to the different MDCK strains, with a directory of where to buy them. The present work focused on chromosome content of MDCK cells, a parameter often disregarded by researchers working with these cells.

**Findings:**

Using a general and reliable method for obtaining high yield of metaphasic preparations, the chromosome content of MDCK, MDCK I, MDCK II obtained from reliable sources was determined after maintaining them in culture for various periods of time. Within two months significant changes were observed in the range and the mean number of chromosomes of MDCK I and MDCK II cells. MDCK II cells routinely cultured in six different laboratories were also examined. In some of these cultures the cells have considerably drifted as shown by a high scattering in their number of chromosomes.

**Conclusion:**

These results entitle me to encourage researchers using MDCK cells obtained from reliable sources, to determine their chromosomal content upon receipt, to check this content after several passages, to use this feature to follow the possible drift of these cells, and finally to avoid working with cells maintained for more than two months in culture.

## Findings

### Background

A great variety of cell lines are used to study numerous fields of cell biology. Very few of these lines present a normal karyotype. They are generally aneuploid and often contain rearranged chromosomes. Moreover, depending on the duration and the conditions of culture, populations of these cell lines can change. Because of possible drift, cell lines need to be regularly characterized and checked, one fundamental verification being to test the stability of their chromosome content. Unfortunately, this is often disregarded by researchers working with cell lines, as exemplified here for MDCK cells.

MDCK cells [1] constitute one of the best models for studying protein trafficking, polarity and junctions in epithelial cells (over 6600 references in PubMed [2]). As reported by Dukes et al. [3], researchers often report on "MDCK cells" with no reference to their strain nor to the supplier. Moreover, among the studies on MDCK, very few (4 references in PubMed [4]) have payed attention to the chromosome content of these cells (or that of their subclones [5]) and none had examined the possible drift of this content during the culture.

In the present work the chromosome content of MDCK, MDCK I, MDCK II from different sources was determined at different passages and/or generations. Significant and even large differences were observed in the range and the mean number of chromosomes.

### Methods

**Figure 1:**
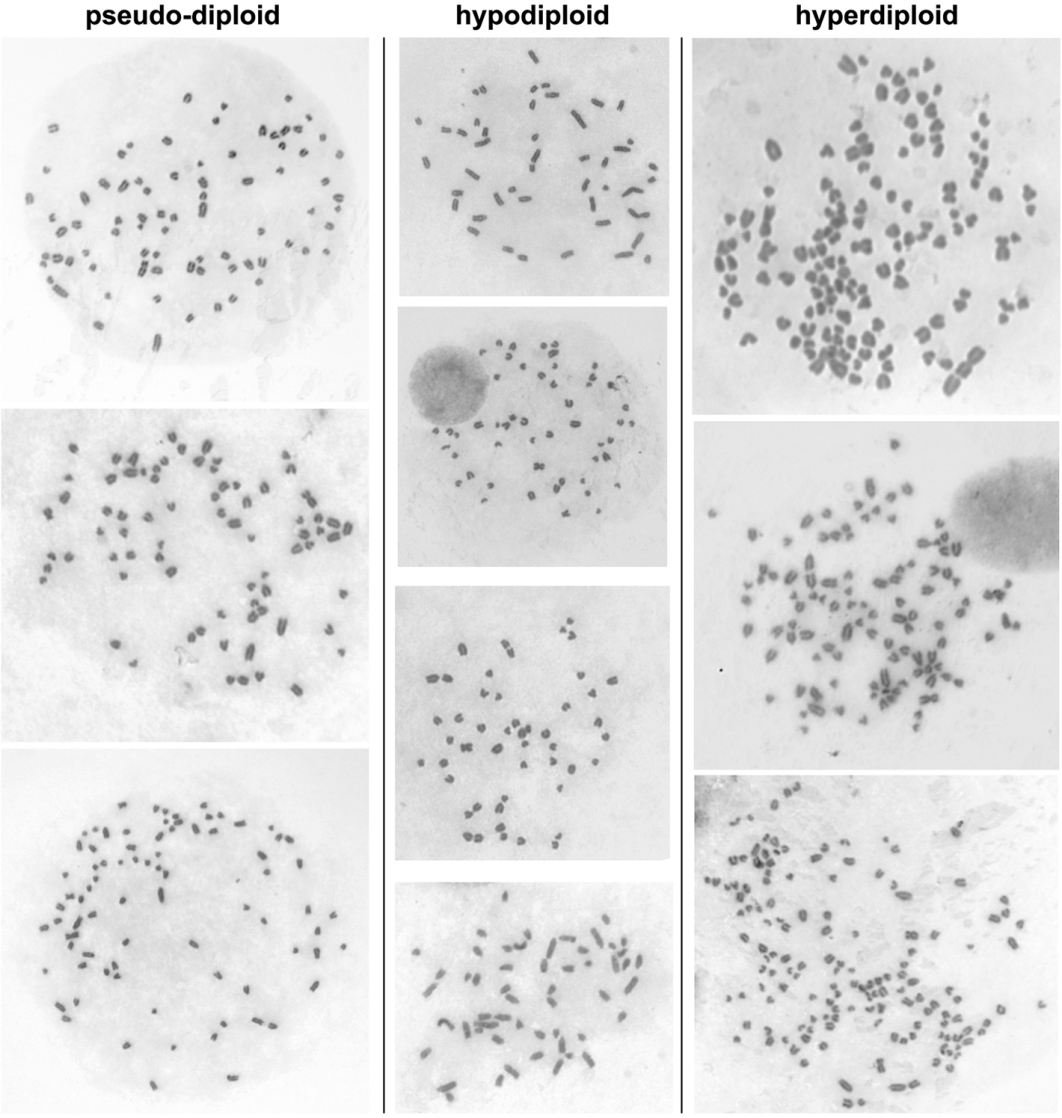
**Metaphases of MDCK II cells.** Pseudo-diploid metaphases (left) were from cells maintained one month in culture. Hypodiploid metaphases (middle) were from cells maintained 2-3 months in culture. Hyperdiploid metaphases (right) were from top to bottom from the MDCKII cultures shown in Figure 3F, C, E, respectively.

The parental MDCK (NBL-2) line and the strains MDCK I and MDCK II, were obtained from the American Type Culture Collection (ATCC; [http://www.atcc.org]), Kai Simons [6] and the European Collection Cell cultures (ECACC; [http://www.hpacultures,org,uk/collections]) (Table 1). MDCK II cells were also obtained from six different laboratories, where these cells are routinely cultured. Cells were cultured as previously described in F-12 Coon’s modified medium (Sigma-Aldrich) supplemented with 5% fœtal calf serum [7]. MDCK II cells (from ECACC) were also cultured in DMEM medium. The changes of chromosome content were similar in both media. Cells were plated at 2-4 10^3^ cells/cm^2^ and passed every 5-7 days; MDCK II was also passed twice a week. At each passage, cells were counted and the number of generations calculated; one passage every 5-7 days corresponded to 5-7 generations. Metaphases were prepared from cultures in exponential phase, using a method giving a high yield in metaphases [8]. This method is easy, reliable and suitable metaphases can be obtained and then counted rapidly. It has been used before to evaluate the mean number and the range of chromosomes of many lines (L(Cl1D), WI38, V79-4, HT29, Fao, BW1-J) [8]. The results were similar to those previously published, showing the reliability of this method. The Giemsa solid staining [9] which gives uniform staining of the chromosomes and makes it easy to count them was used.. The mean number of chromosomes and the range were obtained by analyzing 50 metaphases. In the case of very heterogeneous cultures 85 metaphases were examined. The number of chromosomes of each metaphase was counted twice. Examples of metaphases of MDCK II are shown in Figure 1.

**Table 1:** Chromosome content of MDCK, MDCK I, and MDCK II cells

**Strain**	**Supplier**	**Passage, time in culture**	**Chromosome mean number**	**Range**	**Figure**
**MDCK**	ATCC	passage 11 (upon receipt)	77*	74-81	Figure 1A
		- maintained for two months in culture (10 passages, 60 generations)	75	68-81	Figure 1B
**MDCK I**	Kai Simmons	passage 21 (upon receipt)	79*	76-83	Figure 1C
		- maintained for one month in culture (5 passages, 30 generations)	78	72-82	Figure 1D
		- maintained for two months in culture (10 passages, 60generations)	76	60-80	Figure 1E
**MDCK II**	ECACC	passage 27 (upon receipt)	75*	69-80	Figure 1F
		- maintained for one month in culture (5 passages, 30 generations)	75	66-82	Figure 1G
		- maintained for two months in culture (10 passages, 60 generations)	73	52-81	Figure 1H
		- maintained for two months in culture (20 passages, 60 generations)	73	51-81	Figure 1I
		- maintained for three months in culture (15 passages, 90 generations)	69	47-82	Figure 1J
**MDCK II**	from six different laboratories	passage 46	78	74-81	Figure 2A
		passage unknown	75*	67-82	Figure 2B
		passage unknown	75	63-105	Figure 2C
		passage 16	71	51-79	Figure 2D
		passage 26	78 ^a^	49-157	Figure 2E
		passage unknown	112^a^	60-144	Figure 2F

* mean number and modal number, ^a^ 85 metaphases analyzed.

### Chromosome content of MDCK, MDCK I, MDCK II and evolution of this content with time

**Figure 2:**
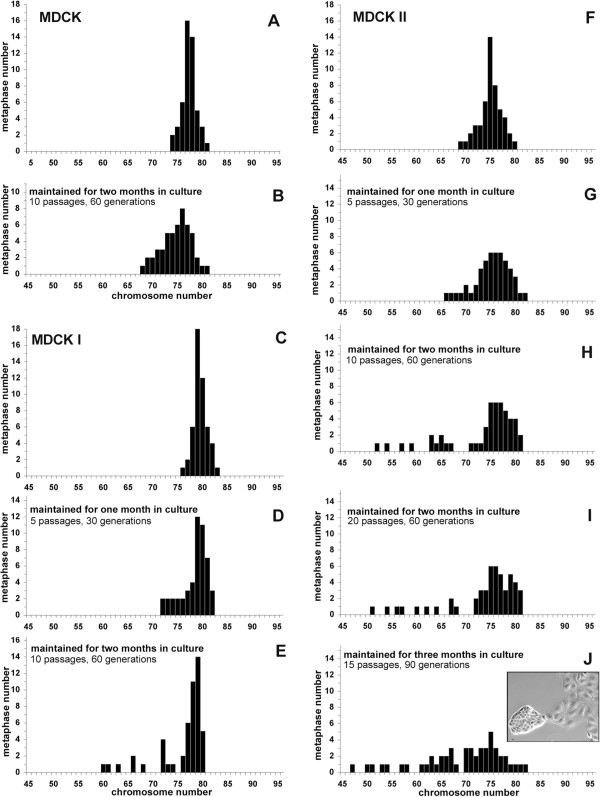
**Analysis, and evolution with time in culture, of metaphases of MDCK (A,B), MDCK I (C-E) and MDCK II (F-J) cells.** Cells obtained from reliable sources (Table 1) were maintained continuously in culture for 2-3 months. The number of chromosomes of 50 metaphases was determined upon their receipt and after various periods of time in culture. Insert in J: phase contrast image of cells maintained for three months in culture. Note the heterogeneity of the cell population, composed of two types of cells: large and flat cells on the right and small and tight cells on the left. This latter type was observed only after two months in culture. Bar, 10 μm.

The pseudo-diploid parental MDCK (NBL-2) line and its two subclones MDCK I and MDCK II were obtained at early passages from reliable suppliers (Table 1). The chromosome content was determined upon receipt of the cells, and after several passages (Table 1, Figure 2). For MDCK cells, as for MDCK I and MDCKII, the chromosome content of early cultures was similar to that previously published [5,6,8]. The range was narrow and the mean number of chromosomes coincided with the modal number (Table 1). No change was observed for the parental MDCK cells after two months in culture (Table 1, Figure 2A,B). In contrast, changes were noticed as MDCK I and MDCK II cells were maintained in culture for months (Figure 2C-E; Figure 2F-J). The populations became more heterogeneous, the mean number of chromosomes decreased and differed from the modal number, and the range was larger (Table 1). Among MDCK II cells maintained for two months, 20% of metaphases had lost some of their chromosomes (compare Figure 2H,I to Figure 2F) and the phenomenon increased in time, as 34% of metaphases had even fewer chromosomes after three months (Figure 2J). It should be noted that the chromosome content of MDCK II cells maintained for two months and 60 generations in culture was not sensitive to the number of passages (compare 10 passages, Figure 2H to 20 passages, Figure 2I). Moreover, MDCK II cell populations became heterogeneous in morphology with time in culture, as attested by the emergence of small cells forming very tight colonies when cells were cultured for three months (insert in Figure 2J). In parallel, MDCK II cell density at confluency significatively changed from 0.9 10^5^ cells/cm^2^ (early cultures) to 1.4 10^5^ cells/cm^2^ (three-month culture) (p = 0.005). The tendency to select cells with fewer chromosomes as culture lasts longer was also observed for MDCK I (Figure 2C-E).

### Chromosome content of MDCK II cell populations from six different laboratories

**Figure 3:**
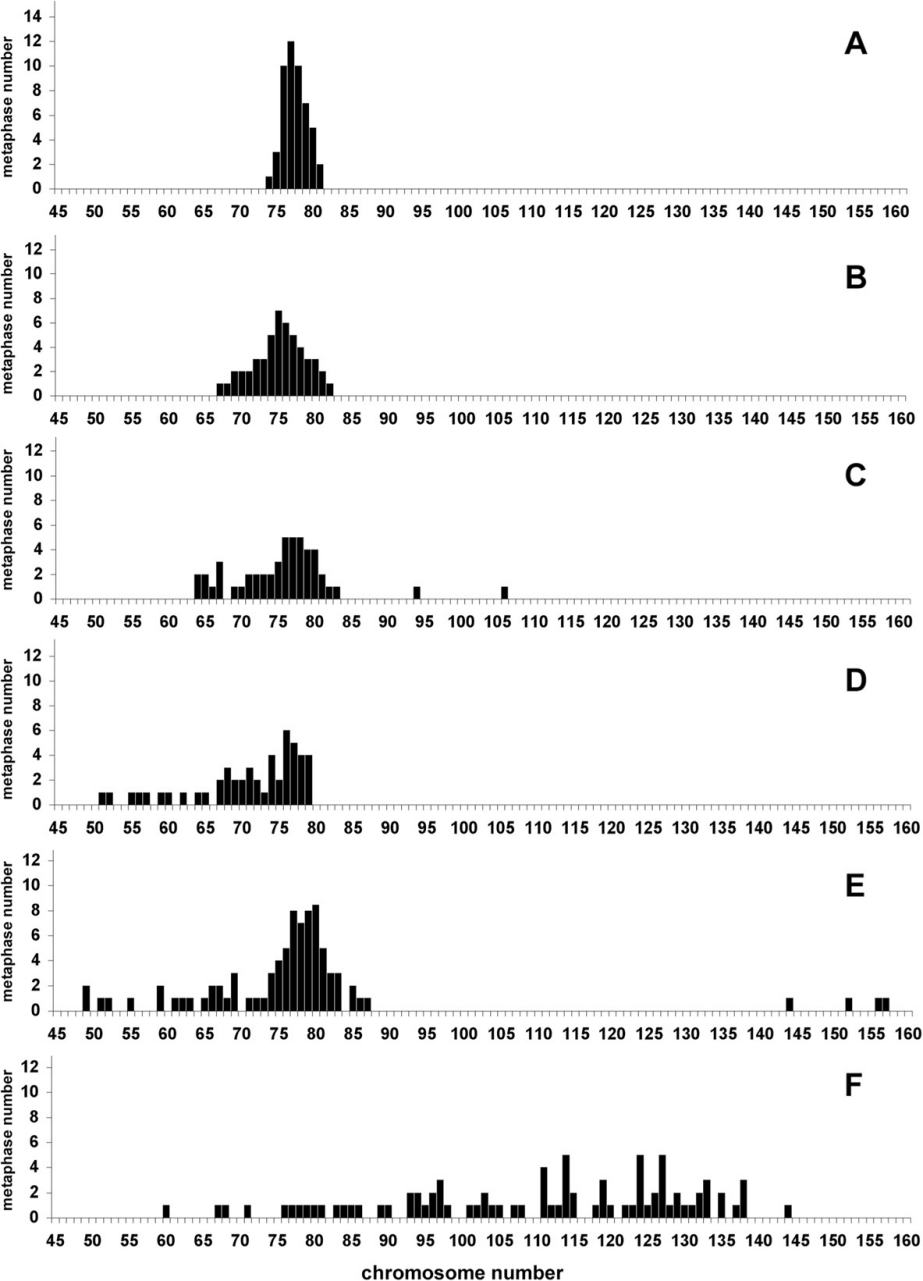
**Analysis of metaphases of MDCK II cells obtained from six different laboratories (A-F).** Cells were provided by laboratories working routinely with MDCK II cells. The number of chromosomes of 50-85 metaphases (Table 1) of these different MDCK cell populations was determined upon their receipt. The results are presented according to the drift of the population, starting from the most homogeneous one.

MDCK II is by far the most used MDCK strain. Therefore the chromosome content of MDCK II routinely cultured in six different laboratories was examined (Table 1, Figure 3). These cells were kindly given for the purpose of this study, but for most of them, no information on the supplier and/or on passage number was available. Moreover when the passage number was known, it was difficult to determine the real age of the culture, because the conditions such as the plating density at each passage, the time interval between two passages, and more importantly, the number of generations per passage, differed from one laboratory to another.

Striking differences in the chromosome content were observed between the six populations examined. Two of them (Figure 3A,B) were similar to the culture of MDCK II provided by ECACC (Figure 1F) (similar mean number and range). The four others had become more heterogeneous (Figure 3C-F), and even though the mean number of chromosomes was not far from the expected number (Table 1), the range was wider, reflecting the presence of metaphases with a low number of chromosomes (Figure 3C-F). In some cases, few metaphases with a high number of chromosomes (Figure 3E-F) were present. One population was very different as it was particularly heterogeneous, displaying a wide range of number of chromosomes (60-144) and 81% of metaphases containing more than 90 chromosomes (Figure 3F).

### Discussion

The present work aimed at studying the chromosome content of MDCK, MDCK I and MDCK II strains. To ensure that the metaphase preparations were representative of the whole cell populations, several precautions were taken. First, I used a method giving a high yield of metaphases [8]. Second, the fixation steps and the spreading of metaphases were carefully carried out to avoid broken and therefore incomplete metaphases. Finally chromosome counting was performed on round metaphases with no or few overlapping chromosomes and that were still embedded with a little of cytoplasm (Figure 1). The embedding decreased the risk of chromosome loss during the spreading.

The data presented here illustrate the fact that cell populations can considerably drift. The main change observed for MDCK I and MDCK II cells maintained for a long time in culture, was the emergence of an increasing fraction of cells with fewer chromosomes. This phenomenon has been previously reported for other cell lines, in particular for HeLa cells [8]. It is very likely due to the fact that cells with a lower chromosomal content grow more rapidly and prevail on cells with more chromosomes. Another change observed in some MDCK II cell populations (Figure 2E,F) was the presence of few metaphases with a higher number of chromosomes. Such metaphases could result from endoreplication events [10], leading to the generation of cells, initially containing twice as much chromosomes, but eventually losing some over generations.

The number of passages is a parameter often used for characterizing a cell line. Unfortunately in most cases information on the passage itself is missing, in particular the number of generations per passage. Therefore, even if cells are examined at the same passage, they could differ by far. For instance MDCK cells examined at a given passage did not have the same efficiency in forming tumors [11]. Moreover, as reported here, the chromosome contents of MDCK II cells examined at similar passages were very different (compare Figure 2F to Figure 3E). This is the reason why it is highly preferable to evaluate the age of a culture in generations and to compare the results obtained at the same generation, rather than at the same passage.

### Conclusions

The present study illustrates for MDCK strains, one fundamental issue encountered in maintaining cells in culture, namely the potential drift of the cell population over time. This point is most often disregarded, or even ignored. Even though it is impossible to prevent such a drift, precautions can be taken to limit the consequences of the drift. First it is highly recommended to obtain cell lines from reliable sources rather than from laboratories working with these lines, except if these laboratories can give information on the source, the number of generations (or passages), the chromosome content and the homogeneity of their cell populations. Second, upon receipt of the cells, a frozen stock of several vials has to be constituted. The chromosome content of these cells needs to be established and further checked after several generations (or passages). Finally, even if there are no important changes in the number and the range of chromosomes over time, I strongly recommend to avoid working with cells maintained for more than two months in culture. One should rather go back to the original frozen stock of cells created upon their receipt. As illustrated in the review of Hughes et al. [12], the use of over-passed cell cultures could lead to erroneous and spurious results.

## Abbreviations

MDCK: Madin-Darby canine kidney.

## Competing interest

The author indicates no potential competiting interest.
